# Reporting of structural population assumptions in prevalence-based studies of animal feeding operations and community health

**DOI:** 10.3389/fpubh.2026.1815580

**Published:** 2026-05-22

**Authors:** Brayan Alexander Fonseca Martinez, Jan M. Sargeant, Sarah C. Totton, Chong Wang, Annette M. O’Connor

**Affiliations:** 1 Department of Large Animal Clinical Sciences, Michigan State University, East Lansing, MI, United States; 2 Department of Population Medicine, Ontario Veterinary College, University of Guelph, Guelph, ON, Canada; 3 Department of Statistics, Iowa State University, Ames, IA, United States; 4 Department of Veterinary Diagnostic and Production Animal Medicine, Iowa State University, Ames, IA, United States

**Keywords:** animal feeding operations (AFOs), causal interpretation, environmental epidemiology, incidence density ratio, prevalence

## Abstract

Research on community health effects of Animal Feeding Operations (AFOs) frequently relies on prevalence-based effect measures, particularly for chronic respiratory outcomes. Interpreting these measures as indicators of comparative disease occurrence requires specific structural population assumptions, yet it remains unclear whether such assumptions are reported in this literature. We conducted a Mini Review of observational studies identified through a previously published systematic review and an ongoing living systematic review to assess whether prevalence studies of AFO exposures and community health explicitly reported the assumptions required to interpret prevalence ratios or prevalence odds ratios as approximations of comparative incidence. Eligible studies used prevalent disease status and reported comparative prevalence-based effect measures. We assessed whether authors discussed assumptions related to population stability, outcome duration, temporal ordering, reverse causality, and disease rarity. Across 15 included studies, none explicitly reported or discussed these structural population assumptions, despite routinely presenting covariate-controlled effect estimates. Greater transparency in reporting population-level assumptions is needed to support valid causal interpretation of prevalence-based effect measures in AFO research and to better inform public health decision-making.

## Introduction

Understanding whether living near Animal Feeding Operations (AFOs) adversely affects the health of surrounding communities remains a complex and highly debated public health issue. Establishing a causal connection would significantly influence public policy and regulatory decisions concerning the siting and emission management of AFOs. However, as is common with environmental issues, establishing a direct causal link poses significant challenges. Studies investigating this topic typically rely on observational methods due to ethical constraints against exposing individuals to potentially harmful conditions (1).

Most studies examining AFO-related community health effects rely on designs that assess chronic health outcomes, such as asthma, using prevalent disease status rather than incident cases. These studies frequently report prevalence ratios (PR) or prevalence odds ratios (POR) to summarize associations (2–5). While these measures are widely used, interpreting prevalence-based contrasts requires attention to key structural features of the source population that determine how prevalence reflects underlying disease occurrence.

Epidemiologic theory has described structural population conditions under which prevalence-based measures may approximate incidence-based measures such as the incidence density ratio (IDR) (6–10). When these conditions are not satisfied, prevalence contrasts may not reflect differences in disease occurrence, potentially complicating causal interpretation. Despite their importance, prior methodological work suggests that these structural assumptions are rarely discussed explicitly in observational environmental health research (11).

Given the extensive use of prevalence-based effect measures in AFO research, it is important to evaluate whether primary studies provide sufficient information about structural population characteristics to interpret the reported results. Reporting these assumptions is essential for transparency and for situating research findings within public health decision-making, especially when prevalence-based effect measures are used to infer potential differences in disease occurrence.

### Objective

The objective of this Mini Review was to synthesize and map how investigators report structural population assumptions when using prevalence-based effect measures in studies of health outcomes among people living near Animal Feeding Operations. Specifically, we addressed the following question: To what extent do studies using prevalent disease status report or discuss the structural population assumptions necessary for interpreting prevalence ratios or prevalence odds ratios as approximations of the incidence density ratio?

## Methods

### Review approach

We conducted a focused methodological synthesis drawing on studies identified through a previously published systematic review (5) and an ongoing living systematic review covering the period 2017–2024. The full protocol describing eligibility criteria and screening procedures is publicly available.^1^ This approach allowed us to examine reporting practices across a well-defined body of literature without generating new effect estimates. Consistent with the aims of a Mini Review, our focus was on conceptual and reporting patterns rather than on quantitative synthesis or assessment of causal effects. Accordingly, we did not perform risk-of-bias assessments or meta-analysis. We use the term *prevalence study* rather than *cross-sectional study* to reflect the analytic focus of the included literature. Many studies measured health outcomes on a cross-section of the population but related these outcomes to exposure measures obtained at different time points, often prior to outcome assessment.

### Information sources and study selection

The literature base for this Mini Review was drawn from two complementary sources addressing the relationship between AFO exposure and community health:

- Systematic review (2014–2017): conducted and published previously (5), identifying observational studies on AFO exposure and community health.
- Living systematic review (2017–2024): updated quarterly beginning in 2021, covering the same topic area and using harmonized methodology.

Both review efforts used harmonized search strategies and eligibility criteria focused on observational studies of AFO exposure and human health outcomes. No additional database searches were conducted specifically for this Mini Review. Instead, all records retrieved through these prior efforts were screened against eligibility criteria developed *a priori* for the present analysis.

### Eligibility criteria

Studies were eligible for inclusion if they met the following criteria:

- Reported an observational study assessing the association between exposure to animal feeding operations and health outcomes among surrounding community members;
- Measured health outcomes at the individual human level;
- Used prevalent disease status to assess at least one health outcome;
- Examined animal feeding operations reasonably characterized as large, concentrated, or intensive by modern standards; and
- Were available in English.

Studies were also excluded if they focused exclusively on occupational exposures, non-human outcomes, nomadic or smallholder agricultural systems, or did not report comparative measures of association. To be included, studies were required to meet all inclusion criteria; studies meeting any of the exclusion criteria were removed during screening.

Finally, for the reporting assessment in this mini-review, only studies presenting comparative associations using prevalence-based effect measures (PR or POR) were assessed. Studies that assessed the associations between AFO and health but reported associations using other metrics (e.g., regression coefficients or mean differences) were excluded from this assessment, as the population structural assumption (i.e., steady-state assumptions) applies only to prevalence-based measures such as PR or POR.

### Study selection

Study selection occurred in two stages. First, titles and abstracts were screened for relevance using DistillerSR® (Evidence Partners, Ottawa, ON, Canada). Second, full-text articles were assessed for eligibility. Screening at both stages was conducted independently by two reviewers (ST and BAFM), with discrepancies resolved through discussion. A calibration exercise was conducted between the two reviewers using a sample of 300 references to ensure consistent interpretation of the eligibility criteria. Following this pretest, agreement between reviewers exceeded 80% across the screening questions used in the two-stage screening process. Agreement between reviewers during screening was subsequently 100% as all conflicts were resolved via discussion or, when consensus could not be reached, a third reviewer (AMOC) adjudicated.

### Data extraction

Data extraction was performed using standardized forms developed and pilot-tested within DistillerSR^®^. Two reviewers independently extracted data from each included study, with disagreements resolved through discussion or third-party adjudication. Extracted information included: Year and location of the study, characteristics of the surrounding community, exposure and health outcomes assessed, and the prevalence-based effect measures reported (PR or POR). Health outcomes were categorized by affected body system, including lower respiratory, upper respiratory, infectious, and other outcomes. All data were extracted exclusively from the published articles; no attempts were made to contact study authors for clarification or additional information. When relevant information was not reported, this was recorded as “not reported”.

### Assessment of structural population assumptions

For each included study, we assessed whether authors explicitly reported or discussed the structural population assumptions required to interpret prevalence-based effect measures as approximations of comparative incidence. For studies that reported a prevalence odds ratio for a common health outcome (i.e., an outcome with an expected prevalence greater 10%), our inquiry focused on whether authors discussed the following assumptions derived directly from Reichenheim and Coutinho (10):

- That the source population is in a steady state over the “study period”
- That the mean duration of the outcome is the same across exposure groups (i.e., independent of exposure status)
- That the outcome cannot cause the exposure status in any way (i.e., no reverse causality)
- That the temporal ordering is correct, such that exposure precedes the outcome

For prevalence studies that reported a prevalence ratio, in addition to evaluating the assumptions, it is necessary for the health event to be rare (i.e., prevalence less than 10%) for these measures to approximate the incidence density ratio (IDR). Our assessment focused solely on reporting and discussion of these assumptions, not on whether the assumptions were likely to hold in practice.

### Synthesis of results

Findings were synthesized descriptively and narratively. We summarized the distribution of study characteristics, the prevalence-based effect measures used, and the extent to which each structural population assumption was reported or discussed. Emphasis was placed on identifying patterns of consistent reporting, partial acknowledgment, or omission of assumptions across the literature.

## Results

Across the literature identified through the parent systematic review and its subsequent updates, 15 studies examining associations between exposure to Animal Feeding Operations (AFOs) and community health outcomes met the inclusion criteria for this Mini Review (12–26). These studies constitute the core body of evidence on which reporting practices regarding prevalence-based effect measures were examined.

Research activity was concentrated in a limited number of high-income countries and focused predominantly on chronic respiratory outcomes, particularly lower respiratory tract conditions. Detailed study characteristics and extracted exposure–outcome pairs are provided in Table 1 and the Supplementary Material. Eleven studies utilized logistic regression for their analysis, making the prevalence odds ratio (POR) the primary reported effect measure without further interpretation regarding their relation to disease incidence (13–16, 19–25). One study employed random-intercepts binary regression, resulting in the extraction of 58 prevalence ratios (PR) (18). Three studies assessed relevant exposure–outcome associations but reported results using regression coefficients or mean differences rather than prevalence-based effect measures; therefore, they did not contribute contrast metrics for which the assumptions related to population steady state would apply (12, 17, 26). Notably, all extracted prevalence-based effect estimates (PR and POR) were reported as covariate-adjusted measures, indicating a consistent effort to control for confounding and implicitly suggesting an interest in etiologic interpretation rather than purely descriptive prevalence comparisons.

**Table 1:** Characteristics of prevalence studies examining associations between exposure to animal feeding operations (AFOs) and community health outcomes.

Study	Country	Population studied	Health outcome category	Effect measure used	Number of exposure–outcome pairs
(13)	USA	Rural Iowa veterans admitted to the Iowa City Veterans Affairs Health Care System	Antimicrobial resistance	Prevelence odds ratio	2
(15)	The Netherlands	Adults living in the east of North-Brabant and the north of Limburg	Lower respiratory	Prevelence odds ratio	10
(16)	Germany	Children aged 5–6 years in an intensive agricultural region of Lower Saxony	Lower respiratory	Prevelence odds ratio	8
(18)	USA	Adolescent students in North Carolina	Lower respiratory	Prevalence ratio	58
(14)	Mexico	Children aged 3–16 year in the municipalities of Amecameca and Chalco	Infectious conditions	Prevelence odds ratio	1
(19)	The Netherlands	Patients registered with general practitioners in Noord Brabant and Limburg	Lower respiratory	Prevelence odds ratio	6
(21)	Germany	Adults living in rural towns with a high density of confined animal feeding operations	Lower respiratory	Prevelence odds ratio	2
			Upper respiratory		2
(20)	Germany	Adults living in rural towns with a high density of confined animal feeding operations	Lower respiratory	Prevelence odds ratio	8
			Upper respiratory		2
(22)	USA	Rural adults, non-farming residents of Wisconsin	Lower respiratory	Prevelence odds ratio	6
			Upper respiratory		2
(23)	Germany	Adults aged 18–44 years living in a rural town in Lower Saxony	Lower Respiratory	Prevelence odds ratio	1
			Upper respiratory		2
(25)	The Netherlands	Patients attending general practices in Noord-Brabent and Limburg	Lower respiratory	Prevelence odds ratio	2
			Infectious conditions		2
(24)	The Netherlands	Patients attending general practices in Noord-Brabent and Limburg	Lower respiratory	Prevelence odds ratio	14
			Upper respiratory		5

### Reporting of structural population assumptions

Despite the common reporting of adjusted prevalence-based effect measures (PR or POR) in 12 of 15 studies, none of the included studies reported or discussed any of the structural population assumptions required to interpret prevalence-based effect measures as estimates of comparative incidence. Specifically, no study addressed assumptions related to population steady state, equality of mean disease duration across exposure groups, absence of reverse causation, or correct temporal ordering between exposure and outcome. For the rare disease assumption, which is required when authors report prevalence ratios or prevalence odds ratios as approximations of the incidence density ratio, this assumption was not applicable in 14 studies because the health outcomes examined were common (>10% prevalence), and was not met in the remaining study.

## Discussion

Across 12 of 15 prevalence studies identified that reported adjusted PR or adjusted POR, none explicitly reported or discussed the structural population assumptions required to interpret prevalence ratios or prevalence odds ratios as indicators of comparative incidence, despite routinely presenting covariate-adjusted effect estimates. This omission is notable because the consistent use of covariate control implicitly signals an interest in causal interpretation, rather than a purely descriptive comparison of disease prevalence (27). Although cohort designs are generally considered the most appropriate observational approach for estimating incidence contrasts when randomized trials are infeasible, epidemiologic theory has long recognized that prevalence studies may, under specific conditions, approximate incidence density ratio (6, 8–10). These conditions relate to structural features of the source population, including population stability, absence of reverse causation, appropriate temporal ordering, and assumptions about outcome duration. Importantly, these requirements do not depend on statistical modeling choices, but on how disease occurrence and population dynamics operate in the underlying population.

Despite the availability of this theoretical framework, none of the studies reviewed discussed whether these conditions were plausibly met for the populations and outcomes under investigation. This gap limits readers’ ability to determine whether reported prevalence odds ratios or prevalence ratios should be interpreted as measures of disease occurrence or as cross-sectional associations only. As emphasized by Rothman and colleagues, such assumptions “rarely, if ever, provide a secure basis for studying prevalence as a proxy for incidence” unless they are explicitly considered and justified (11). Several structural assumptions are particularly challenging to assess in prevalence studies, including temporal ordering and reverse causation. For chronic conditions with long disease duration, demonstrating that exposure preceded outcome requires more than showing that exposure was measured before outcome assessment; it requires justification that exposure occurred before disease onset. Similarly, while reverse causation may be unlikely in some residential exposure contexts, selective migration related to health status cannot be ruled out without explicit consideration. These issues underscore the importance of authors explicitly addressing population dynamics, exposure timing, and disease natural history when interpreting prevalence-based estimates. Doing so would not require additional data collection, but rather clearer articulation of assumptions and their plausibility in the study context.

From an evidence synthesis perspective, the lack of reporting on structural assumptions complicates efforts to compare and interpret findings across studies. Systematic reviews and policy assessments often rely on prevalence-based estimates as indicators of health risk, yet without clear guidance from primary studies, causal interpretation remains uncertain. Improved reporting would allow reviewers and decision-makers to better contextualize prevalence-based evidence, distinguish descriptive from etiologic findings, and identify where incidence-based or longitudinal data are most needed (28).

Several factors may explain the limited reporting of structural population assumptions in the studies reviewed. In environmental epidemiology, prevalence-based measures are frequently used to describe the distribution of health outcomes within populations rather than to approximate incidence-based causal contrasts. When the primary objective is descriptive, such as estimating the burden of disease, identifying potentially affected communities, or informing public health planning and surveillance, authors may not consider it necessary to discuss the structural conditions required for etiologic interpretation of prevalence-based effect measures. It is also possible that some investigators implicitly assume these conditions without explicitly stating them, particularly in settings where environmental exposures are conceptualized as relatively stable population characteristics.

While the absence of explicit discussion of structural assumptions limits the utility of PR and POR estimates for causal inference, it should not be interpreted as diminishing the scientific value of descriptive prevalence studies. Estimates of PR and POR obtained from such studies play a critical role in public health by characterizing the distribution and burden of disease, identifying vulnerable populations, generating hypotheses, and informing resource allocation and policy decisions (28). However, improving transparency in the reporting of structural population assumptions in environmental epidemiology would enable clearer causal inference statements to be made and therefore strengthen the interpretability of evidence used to guide environmental regulation and land-use planning decisions. In the context of AFOs siting and community exposure concerns, clearer articulation of how prevalence-based measures relate to disease occurrence may enhance the reliability of evidence informing public health protection strategies. By promoting more rigorous interpretation of environmental health research, this work contributes to advancing Sustainable Development Goals 3 (good health and well-being) and supports sustainable community health decision-making.

Our findings underscore a broader issue in environmental epidemiology: prevalence-based analyses are frequently interpreted in ways that imply causal inference, yet the assumptions necessary for such interpretations are rarely articulated. While authors of primary studies are best positioned to evaluate population stability, migration patterns, and exposure timing, failure to report these considerations leaves uncertainty at the evidence synthesis and policy interpretation stages (29, 30). Existing reporting guidelines, such as Strengthening the Reporting of Observational Studies in Epidemiology (STROBE) (31), emphasize clarity in study design and timing but do not explicitly address the structural assumptions required for interpreting prevalence-based effect measures as incidence parameters (32). As noted in reporting guidance for observational studies, variants of standard observational designs require additional clarity regarding population definition, timing, and interpretation. When authors seek to make causal inferences, implicitly or explicitly, transparent discussion of structural population assumptions becomes especially important.

This review has several limitations that should be considered when interpreting the findings. First, the search was restricted to studies published in English, which may have excluded relevant research conducted in other languages. Second, although screening and data extraction were performed using predefined criteria and calibration exercises to ensure consistency between reviewers, some degree of subjective judgment may remain inherent in evaluating whether studies explicitly addressed structural population assumptions. Third, the review focused specifically on studies examining community health effects of exposure to animal feeding operations. While this focus allowed a detailed examination of a well-defined body of environmental epidemiologic literature, it may limit the generalizability of the findings to other environmental exposure contexts. Nevertheless, the conceptual issues related to interpreting prevalence-based effect measures and the articulation of structural population assumptions are not unique to AFO-related research and may be relevant across environmental epidemiology more broadly.

## Conclusion

Prevalence studies may, under specific conditions, provide estimates that approximate comparative incidence and thus contribute to causal understanding of the health effects associated with Animal Feeding Operations. However, current reporting practices in this literature limit that possibility. In the studies reviewed, none explicitly addressed the structural population assumptions required to interpret prevalence-based effect measures as incidence parameters. We recommend that authors conducting prevalence studies of environmental exposures clearly state the intended interpretation of their effect measures and explicitly discuss the assumptions under which such interpretations would be valid. Greater conceptual transparency would strengthen the interpretability, comparability, and public health relevance of observational evidence on AFO exposures and community health.

Future methodological guidance could strengthen the interpretability of prevalence-based analyses in environmental epidemiology by encouraging authors to explicitly report key structural population assumptions when prevalence ratios or prevalence odds ratios are interpreted in relation to disease occurrence. Such reporting could include consideration of population stability over the study period, the temporal relationship between exposure and outcome, and whether outcome status could influence exposure classification. Incorporating these elements into reporting guidance or complementary extensions to existing frameworks such as the STROBE could improve transparency and support more consistent interpretation of environmental health evidence used to inform public health and policy decisions.

## Footnotes

1
https://syreaf.org/wp-content/uploads/2024/03/Job-2-Protocol-for-Prevalance-scoping-review.pdf
