# Supplementary material for: Reporting of structural population assumptions in prevalence-based studies of animal feeding operations and community health
Source: Front Public Health. 2026 May 22;14:1815580. doi: 10.3389/fpubh.2026.1815580 (PMC13236910; doi:10.3389/fpubh.2026.1815580)
Supplement: Supplementary file 1 [file Table_1.docx]

**Supplementary Table S1.**

Exposure–outcome pairs extracted from prevalence studies examining associations between exposure to Animal Feeding Operations (AFOs) and community health outcomes. The table reports all **133 exposure–outcome pairs** identified across the included studies, including the **effect measure used** (prevalence odds ratio or prevalence ratio), the **specific outcome evaluated**, the corresponding **health outcome category**, and the **exposure evaluated**. This table provides the detailed basis for the summary results presented in the main text.

| **Study** | **Effect measure used** | **Outcome evaluated** | **Health outcome category** | **Exposure evaluated** |
| --- | --- | --- | --- | --- |
| Mirabelli et al. 2006 | Prevalence ratio | Current wheeze children with Self-Reported Allergies | Lower Respiratory | Exposure category |
| Mirabelli et al. 2006 | Prevalence ratio | Current wheeze children without Self-Reported Allergies | Lower Respiratory | Exposure category |
| Mirabelli et al. 2006 | Prevalence ratio | Current wheeze in all children | Lower Respiratory | Exposure category |
| Mirabelli et al. 2006 | Prevalence ratio | Current wheeze children with Self-Reported Allergies | Lower Respiratory | Hog pounds (in millions) within 3 miles of school |
| Mirabelli et al. 2006 | Prevalence ratio | Current wheeze children without Self-Reported Allergies | Lower Respiratory | Hog pounds (in millions) within 3 miles of school |
| Mirabelli et al. 2006 | Prevalence ratio | Current wheeze in all children | Lower Respiratory | Hog pounds (in millions) within 3 miles of school |
| Mirabelli et al. 2006 | Prevalence ratio | Current wheeze children with Self-Reported Allergies | Lower Respiratory | Livestock odor |
| Mirabelli et al. 2006 | Prevalence ratio | Current wheeze children without Self-Reported Allergies | Lower Respiratory | Livestock odor |
| Mirabelli et al. 2006 | Prevalence ratio | Current wheeze in all children | Lower Respiratory | Livestock odor |
| Mirabelli et al. 2006 | Prevalence ratio | Current wheeze children with Self-Reported Allergies | Lower Respiratory | Livestock Odor Reported Outside or Inside School Building |
| Mirabelli et al. 2006 | Prevalence ratio | Current wheeze without physician diagnosis in children with self-reported allergies | Lower Respiratory | Livestock Odor Reported Outside or Inside School Building |
| Mirabelli et al. 2006 | Prevalence ratio | Severe wheeze in children with self reported allergies | Lower Respiratory | Livestock Odor Reported Outside or Inside School Building |
| Mirabelli et al. 2006 | Prevalence ratio | Frequent severe wheeze in children with self reported allergies | Lower Respiratory | Livestock Odor Reported Outside or Inside School Building |
| Mirabelli et al. 2006 | Prevalence ratio | Physician diagnosied Asthma in children with self reported allergies | Lower Respiratory | Livestock Odor Reported Outside or Inside School Building |
| Mirabelli et al. 2006 | Prevalence ratio | Asthma medication use in past year self-reported allergies | Lower Respiratory | Livestock Odor Reported Outside or Inside School Building |
| Mirabelli et al. 2006 | Prevalence ratio | Activity limitations in past year as a result of asthma symptoms | Lower Respiratory | Livestock Odor Reported Outside or Inside School Building |
| Mirabelli et al. 2006 | Prevalence ratio | Current wheeze without physician diagnosis in children with no self-reported allergies | Lower Respiratory | Livestock Odor Reported Outside or Inside School Building |
| Mirabelli et al. 2006 | Prevalence ratio | Current wheeze children without Self-Reported Allergies | Lower Respiratory | Livestock Odor Reported Outside or Inside School Building |
| Mirabelli et al. 2006 | Prevalence ratio | Severe wheeze in children with no self reported allergies | Lower Respiratory | Livestock Odor Reported Outside or Inside School Building |
| Mirabelli et al. 2006 | Prevalence ratio | Frequent severe wheeze in chidlren with no self reported allergies | Lower Respiratory | Livestock Odor Reported Outside or Inside School Building |
| Mirabelli et al. 2006 | Prevalence ratio | Physician diagnosied Asthma in children with no self reported allergies | Lower Respiratory | Livestock Odor Reported Outside or Inside School Building |
| Mirabelli et al. 2006 | Prevalence ratio | Asthma medication use in past year no self-reported allergies | Lower Respiratory | Livestock Odor Reported Outside or Inside School Building |
| Mirabelli et al. 2006 | Prevalence ratio | Current wheeze in all children | Lower Respiratory | Livestock Odor Reported Outside or Inside School Building |
| Mirabelli et al. 2006 | Prevalence ratio | Severe Wheeze in all children | Lower Respiratory | Livestock Odor Reported Outside or Inside School Building |
| Mirabelli et al. 2006 | Prevalence ratio | Current wheeze without physician diagnosis in all children | Lower Respiratory | Livestock Odor Reported Outside or Inside School Building |
| Mirabelli et al. 2006 | Prevalence ratio | Frequent severe wheeze in all children | Lower Respiratory | Livestock Odor Reported Outside or Inside School Building |
| Mirabelli et al. 2006 | Prevalence ratio | Physician diagnoses asthma in all children | Lower Respiratory | Livestock Odor Reported Outside or Inside School Building |
| Mirabelli et al. 2006 | Prevalence ratio | Asthma medication use in past year all children | Lower Respiratory | Livestock Odor Reported Outside or Inside School Building |
| Mirabelli et al. 2006 | Prevalence ratio | Current wheeze without physician diagnosis in children with self-reported allergies | Lower Respiratory | >=3 vs <3 Miles From Nearest Swine CAFO |
| Mirabelli et al. 2006 | Prevalence ratio | Severe wheeze in children with self reported allergies | Lower Respiratory | >=3 vs <3 Miles From Nearest Swine CAFO |
| Mirabelli et al. 2006 | Prevalence ratio | Frequent severe wheeze in children with self reported allergies | Lower Respiratory | >=3 vs <3 Miles From Nearest Swine CAFO |
| Mirabelli et al. 2006 | Prevalence ratio | Physician diagnosied Asthma in children with self reported allergies | Lower Respiratory | >=3 vs <3 Miles From Nearest Swine CAFO |
| Mirabelli et al. 2006 | Prevalence ratio | Activity limitations in past year as a result of asthma symptoms | Lower Respiratory | >=3 vs <3 Miles From Nearest Swine CAFO |
| Mirabelli et al. 2006 | Prevalence ratio | Asthma medication use in past year self-reported allergies | Lower Respiratory | >=3 vs <3 Miles From Nearest Swine CAFO |
| Mirabelli et al. 2006 | Prevalence ratio | Current wheeze without physician diagnosis in children with no self-reported allergies | Lower Respiratory | >=3 vs <3 Miles From Nearest Swine CAFO |
| Mirabelli et al. 2006 | Prevalence ratio | Severe wheeze in children with no self reported allergies | Lower Respiratory | >=3 vs <3 Miles From Nearest Swine CAFO |
| Mirabelli et al. 2006 | Prevalence ratio | Frequent severe wheeze in chidlren with no self reported allergies | Lower Respiratory | >=3 vs <3 Miles From Nearest Swine CAFO |
| Mirabelli et al. 2006 | Prevalence ratio | Physician diagnosied Asthma in children with no self reported allergies | Lower Respiratory | >=3 vs <3 Miles From Nearest Swine CAFO |
| Mirabelli et al. 2006 | Prevalence ratio | Asthma medication use in past year no self-reported allergies | Lower Respiratory | >=3 vs <3 Miles From Nearest Swine CAFO |
| Mirabelli et al. 2006 | Prevalence ratio | Current wheeze without physician diagnosis in all children | Lower Respiratory | >=3 vs <3 Miles From Nearest Swine CAFO |
| Mirabelli et al. 2006 | Prevalence ratio | Severe Wheeze in all children | Lower Respiratory | >=3 vs <3 Miles From Nearest Swine CAFO |
| Mirabelli et al. 2006 | Prevalence ratio | Frequent severe wheeze in all children | Lower Respiratory | >=3 vs <3 Miles From Nearest Swine CAFO |
| Mirabelli et al. 2006 | Prevalence ratio | Physician diagnoses asthma in all children | Lower Respiratory | >=3 vs <3 Miles From Nearest Swine CAFO |
| Mirabelli et al. 2006 | Prevalence ratio | Asthma medication use in past year all children | Lower Respiratory | >=3 vs <3 Miles From Nearest Swine CAFO |
| Mirabelli et al. 2006 | Prevalence ratio | Current wheeze children with Self-Reported Allergies | Lower Respiratory | Miles to nearest swine CAFO |
| Mirabelli et al. 2006 | Prevalence ratio | Current wheeze children without Self-Reported Allergies | Lower Respiratory | Miles to nearest swine CAFO |
| Mirabelli et al. 2006 | Prevalence ratio | Current wheeze in all children | Lower Respiratory | Miles From Nearest Swine CAFO |
| Mirabelli et al. 2006 | Prevalence ratio | Current wheeze children with Self-Reported Allergies | Lower Respiratory | >=3 vs <3 Miles From Nearest Swine CAFO |
| Mirabelli et al. 2006 | Prevalence ratio | Current wheeze children without Self-Reported Allergies | Lower Respiratory | >=3 vs <3 Miles From Nearest Swine CAFO |
| Mirabelli et al. 2006 | Prevalence ratio | Current wheeze in all children | Lower Respiratory | >=3 vs <3 Miles From Nearest Swine CAFO |
| Mirabelli et al. 2006 | Prevalence ratio | Asthma-related physician visit emergency visit and/or hospitalization in past year no self-reported allergies | Lower Respiratory | Livestock Odor Reported Outside or Inside School Building |
| Mirabelli et al. 2006 | Prevalence ratio | Asthma-related physician visit emergency visit and/or hospitalization in past year all children | Lower Respiratory | Livestock Odor Reported Outside or Inside School Building |
| Mirabelli et al. 2006 | Prevalence ratio | Asthma-related physician visit emergency visit and/or hospitalization in the past year self-reported allergies | Lower Respiratory | >=3 vs <3 Miles From Nearest Swine CAFO |
| Mirabelli et al. 2006 | Prevalence ratio | Missed school in past year as a result of asthma symptoms | Lower Respiratory | >=3 vs <3 Miles From Nearest Swine CAFO |
| Mirabelli et al. 2006 | Prevalence ratio | Asthma-related physician visit emergency visit and/or hospitalization in past year no self-reported allergies | Lower Respiratory | >=3 vs <3 Miles From Nearest Swine CAFO |
| Mirabelli et al. 2006 | Prevalence ratio | Asthma-related physician visit emergency visit and/or hospitalization in past year all children | Lower Respiratory | >=3 vs <3 Miles From Nearest Swine CAFO |
| Mirabelli et al. 2006 | Prevalence ratio | Missed school in past year as a result of asthma symptoms | Lower Respiratory | Livestock Odor Reported Outside or Inside School Building |
| Mirabelli et al. 2006 | Prevalence ratio | Asthma-related physician visit emergency visit and/or hospitalization in the past year self-reported allergies | Lower Respiratory | Livestock Odor Reported Outside or Inside School Building |
| Smit et al. 2014 | Prevalence odds ratio | Chronic obstructive pulmonary disease (COPD) | Lower Respiratory | One or more farms within 500 m |
| Smit et al. 2014 | Prevalence odds ratio | Chronic obstructive pulmonary disease (COPD) | Lower Respiratory | Presence of farm animals within 500 m |
| Smit et al. 2014 | Prevalence odds ratio | Asthma (at least 1 episode in past year) | Lower Respiratory | PM10 emission from farms within 500 m |
| Smit et al. 2014 | Prevalence odds ratio | Asthma (at least 1 episode in past year) | Lower Respiratory | Distance to nearest farm |
| Smit et al. 2014 | Prevalence odds ratio | Asthma (at least 1 episode in past year) | Lower Respiratory | One or more farms within 500 m |
| Smit et al. 2014 | Prevalence odds ratio | Asthma (at least 1 episode in past year) | Lower Respiratory | Presence of farm animals within 500 m |
| Smit et al. 2014 | Prevalence odds ratio | Asthma (at least 1 episode in past year) | Lower Respiratory | PM10 emission from farms within 500 m |
| Smit et al. 2014 | Prevalence odds ratio | Asthma (at least 1 episode in past year) | Lower Respiratory | Distance to nearest farm |
| Smit et al. 2014 | Prevalence odds ratio | Asthma (at least 1 episode in past year) | Lower Respiratory | One or more farms within 500 m |
| Smit et al. 2014 | Prevalence odds ratio | Asthma (at least 1 episode in past year) | Lower Respiratory | Presence of farm animals within 500 m |
| Smit et al. 2014 | Prevalence odds ratio | Allergic rhinitis | Upper Respiratory | PM10 emission from farms within 500 m |
| Smit et al. 2014 | Prevalence odds ratio | Allergic rhinitis | Upper Respiratory | Distance to nearest farm |
| Smit et al. 2014 | Prevalence odds ratio | Allergic rhinitis | Upper Respiratory | One or more farms within 500 m |
| Smit et al. 2014 | Prevalence odds ratio | Allergic rhinitis | Upper Respiratory | Presence of farm animals within 500 m |
| Smit et al. 2014 | Prevalence odds ratio | Chronic obstructive pulmonary disease (COPD) | Lower Respiratory | PM10 emission from farms within 500 m |
| Smit et al. 2014 | Prevalence odds ratio | Chronic obstructive pulmonary disease (COPD) | Lower Respiratory | Distance to nearest farm |
| Smit et al. 2014 | Prevalence odds ratio | Chronic obstructive pulmonary disease (COPD) | Lower Respiratory | Number of farms within 500 m |
| Smit et al. 2014 | Prevalence odds ratio | Asthma (at least 1 episode in past year) | Lower Respiratory | Number of farms within 500 m |
| Smit et al. 2014 | Prevalence odds ratio | Allergic rhinitis | Upper Respiratory | Number of farms within 500 m |
| Bullers 2005 | Other Effect measure |  |  |  |
| Smit et al. 2012 | Prevalence odds ratio | Pneumonia | Lower Respiratory | Number of goats within 5 km |
| Smit et al. 2012 | Prevalence odds ratio | Pneumonia | Lower Respiratory | Presence of farm animals within 1 km |
| Smit et al. 2012 | Prevalence odds ratio | Other infectious disease | Infectious conditions | Presence of farm animals within 1 km |
| Smit et al. 2012 | Prevalence odds ratio | Other infectious disease | Infectious conditions | Number of goats within 5 km |
| Schulze et al. 2011 | Prevalence odds ratio | Allergic rhinitis | Upper Respiratory | Interpolated ammonia exposure Age;19.71 &microg/m |
| Schulze et al. 2011 | Prevalence odds ratio | Sensitization against ubiquitous allergens | Upper Respiratory | Interpolated ammonia exposure Age;19.71 &microg/m |
| Schulze et al. 2011 | Prevalence odds ratio | Wheezing without a cold | Lower Respiratory | Interpolated ammonia exposure Age;19.71 &microg/m |
| Radon et al. 2007 | Prevalence odds ratio | Wheezing Without Cold | Lower Respiratory | Level of Odor Annoyance |
| Radon et al. 2007 | Prevalence odds ratio | Specific IgE to Common Allergens | Lower Respiratory | No. of animal houses within 500 m |
| Radon et al. 2007 | Prevalence odds ratio | Wheezing Without Cold | Lower Respiratory | No. of animal houses within 500 m |
| Radon et al. 2007 | Prevalence odds ratio | Bronchial Hyperresponsiveness to Methacholine | Lower Respiratory | Level of Odor Annoyance |
| Radon et al. 2007 | Prevalence odds ratio | Specific IgE to Common Allergens | Lower Respiratory | Level of Odor Annoyance |
| Radon et al. 2007 | Prevalence odds ratio | Bronchial Hyperresponsiveness to Methacholine | Lower Respiratory | No. of animal houses within 500 m |
| Radon et al. 2007 | Prevalence odds ratio | Allergic rhinitis | Upper Respiratory | Level of Odor Annoyance |
| Radon et al. 2007 | Prevalence odds ratio | Allergic rhinitis | Upper Respiratory | No. of animal houses within 500 m |
| Radon et al. 2007 | Prevalence odds ratio | Physician-Diagnosed Asthma | Lower Respiratory | Level of Odor Annoyance |
| Radon et al. 2007 | Prevalence odds ratio | Physician-Diagnosed Asthma | Lower Respiratory | No. of animal houses within 500 m |
| Radon et al. 2005 | Prevalence odds ratio | Allergic rhinitis | Upper Respiratory | Animal houses within 500m |
| Radon et al. 2005 | Prevalence odds ratio | Allergic rhinitis | Upper Respiratory | Level of Odor Annoyance |
| Radon et al. 2005 | Prevalence odds ratio | Non-cold related rhonchal breathing sounds | Lower Respiratory | Animal houses within 500m |
| Radon et al. 2005 | Prevalence odds ratio | Non-cold related rhonchal breathing sounds | Lower Respiratory | Level of Odor Annoyance |
| Hoopmann et al. 2006 | Prevalence odds ratio | Allergic asthma-Non-atopic parents | Lower Respiratory | Log of the Endotoxin |
| Hoopmann et al. 2006 | Prevalence odds ratio | Allergic asthma-Atopic parents | Lower Respiratory | Log of the Endotoxin |
| Hoopmann et al. 2006 | Prevalence odds ratio | Non-allergic asthma-Non-atopic parents | Lower Respiratory | Log of the Endotoxin |
| Hoopmann et al. 2006 | Prevalence odds ratio | Non-allergic asthma-Atopic parents | Lower Respiratory | Log of the Endotoxin |
| Hoopmann et al. 2006 | Prevalence odds ratio | Asthmatic Pathology-Not-Atopic Parents | Lower Respiratory | Log of the Endotoxin |
| Hoopmann et al. 2006 | Prevalence odds ratio | Asthmatic Pathology-Atopic Parents | Lower Respiratory | Log of the Endotoxin |
| Hoopmann et al. 2006 | Prevalence odds ratio | Asthmatic Pathology | Lower Respiratory | Log of the Endotoxin |
| Hoopmann et al. 2006 | Prevalence odds ratio | IgE | Lower Respiratory | Log of the Endotoxin |
| Carrel et al. 2014 | Prevalence odds ratio | MRSA-positive nares screen | Antimicrobial resistance | More than 1000 swine AU within 1 mile |
| Carrel et al. 2014 | Prevalence odds ratio | MRSA-positive nares screen | Antimicrobial resistance | Any swine AU within 1 mile |
| Freidl et al. 2017 | Prevalence odds ratio | Pneumonia | Lower Respiratory | Presence of any type of farm within a certain distance of residence |
| Freidl et al. 2017 | Prevalence odds ratio | Pneumonia | Lower Respiratory | Presence of farm with minimum amount of animals within 500m of residence |
| Freidl et al. 2017 | Prevalence odds ratio | Pneumonia | Lower Respiratory | Presence of farm with minimum amount of animals within 1000m of residence |
| Freidl et al. 2017 | Prevalence odds ratio | Pneumonia | Lower Respiratory | Presence of farm with minimum amount of animals within 1500m of residence |
| Freidl et al. 2017 | Prevalence odds ratio | Pneumonia | Lower Respiratory | Presence of farm with minimum amount of animals within 2000m of residence |
| Freidl et al. 2017 | Prevalence odds ratio | Pneumonia | Lower Respiratory | Distance (quartiles expressed in meters) between residence and closest farm with minimum 250 poultry |
| Freidl et al. 2017 | Prevalence odds ratio | Pneumonia | Lower Respiratory | Distance (quartiles expressed in meters) between residence and closest farm with minimum 50 goats |
| Freidl et al. 2017 | Prevalence odds ratio | Pneumonia | Lower Respiratory | Number of animals within 1000m of the residence |
| Freidl et al. 2017 | Prevalence odds ratio | Pneumonia | Lower Respiratory | Number of animals within 1000m of the residence |
| Freidl et al. 2017 | Prevalence odds ratio | Pneumonia | Lower Respiratory | Number of farms (any type) within 1000m of residence |
| Cortés et al. 2015 | Prevalence odds ratio | Anti-Toxocara canis antibodies | Infectious conditions | Live near livestock farming |
| Schultz et al. 2019 | Prevalence odds ratio | Nasal allergies | Upper Respiratory | Restricted cubic spline of residential distance to the nearest CAFO |
| Schultz et al. 2019 | Prevalence odds ratio | Lung allergies | Lower Respiratory | Restricted cubic spline of residential distance to the nearest CAFO |
| Schultz et al. 2019 | Prevalence odds ratio | Nasal or lung allergies & current asthma | Lower Respiratory | Restricted cubic spline of residential distance to the nearest CAFO |
| Schultz et al. 2019 | Prevalence odds ratio | Current asthma | Lower Respiratory | Restricted cubic spline of residential distance to the nearest CAFO |
| Schultz et al. 2019 | Prevalence odds ratio | Asthma (at least 1 episode in past year) | Lower Respiratory | Restricted cubic spline of residential distance to the nearest CAFO |
| Schultz et al. 2019 | Prevalence odds ratio | Asthma medication use in the past year | Lower Respiratory | Restricted cubic spline of residential distance to the nearest CAFO |
| Schultz et al. 2019 | Prevalence odds ratio | Current allergies | Upper Respiratory | Restricted cubic spline of residential distance to the nearest CAFO |
| Schultz et al. 2019 | Prevalence odds ratio | Physician-Diagnosed Asthma | Lower Respiratory | Restricted cubic spline of residential distance to the nearest CAFO |
| Zomer et al. 2017 | Other Effect measure |  |  |  |
| Kalkowska et al. 2018 | Other Effect measure |  |  |  |
| Post et al. 2019 | Prevalence odds ratio | Pneumonia | Lower Respiratory | Presence of goat farm near residential address |
| Post et al. 2019 | Prevalence odds ratio | Pneumonia | Lower Respiratory | Presence of a poultry farm near residence |
| Post et al. 2019 | Prevalence odds ratio | Pneumonia | Lower Respiratory | Presence of a chicken farm near residence |
| Post et al. 2019 | Prevalence odds ratio | Pneumonia | Lower Respiratory | Presence of farm with laying hens or parent stock near the residence |
| Post et al. 2019 | Prevalence odds ratio | Pneumonia | Lower Respiratory | Presence of farm with broilers near the residence |
| Post et al. 2019 | Prevalence odds ratio | Pneumonia | Lower Respiratory | Presence of farm with other poultry near the residence |
